# Impact of lenvatinib on renal function: long-term analysis of differentiated thyroid cancer patients

**DOI:** 10.1186/s12885-021-08622-w

**Published:** 2021-08-05

**Authors:** Chie Masaki, Kiminori Sugino, Sakiko Kobayashi, Yoshie Hosoi, Reiko Ono, Haruhiko Yamazaki, Junko Akaishi, Kiyomi Y. Hames, Chisato Tomoda, Akifumi Suzuki, Kenichi Matsuzu, Keiko Ohkuwa, Wataru Kitagawa, Mitsuji Nagahama, Koichi Ito

**Affiliations:** 1 Department of Surgery, Ito Hospital, Tokyo, 150-8308 Japan; 2 Department of Internal Medicine, Keio University School of Medicine, Tokyo, 160-8582 Japan

**Keywords:** Lenvatinib, Advanced thyroid carcinoma, Renal function, eGFR, Proteinuria

## Abstract

**Background:**

Because lenvatinib is well known to induce proteinuria by blocking the vascular endothelial growth factor (VEGF) pathway, renal function is a concern with long-term administration of lenvatinib. The long-term effects of lenvatinib on renal function in patients with advanced differentiated thyroid carcinoma (DTC) were analyzed.

**Method:**

This study involved 40 DTC patients who continued lenvatinib therapy for ≥6 months. Estimated glomerular filtration rate (eGFR) was calculated as an indicator of renal function. The temporal course of eGFR, effects of baseline eGFR on eGFR changes, and factors affecting renal impairment were investigated.

**Results:**

The overall cohort showed sustainable decreases in eGFR, with decreased values of 11.4, 18.3, and 21.0 mL/min/1.73 m^2^ at 24, 36, and 48 months after starting treatment, respectively. No differences in eGFR decrease every 6 months were seen for three groups classified by baseline eGFR ≥90 mL/min/1.73 m^2^ (*n* = 6), < 90 but ≥60 mL/min/1.73 m^2^ (*n* = 26), or < 60 but ≥45 mL/min/1.73 m^2^ (*n* = 8). Grade 3 proteinuria was associated with declines in eGFR (*p* = 0.0283). Long observation period was also associated with decreases in eGFR (*p* = 0.0115), indicating that eGFR may decrease in a time-dependent manner.

**Conclusion:**

Lenvatinib can induce declines in eGFR, particularly with treatment duration > 2 years, regardless of baseline eGFR. Proteinuria is a risk factor for declines in eGFR. Patients who start lenvatinib with better renal function show a renal reserve capacity, prolonging clinical outcomes. Decision-making protocols must balance the benefits of lenvatinib continuation with acceptable risks of harm.

## Introduction

Lenvatinib is an agent that shows strong tumor suppression, targeting multiple receptors including vascular endothelial growth factor receptor (VEGFR)-1 to − 3 [1, 2]. The characteristics of inducing proteinuria and hypertension as shared class effects are also well known [2–5], particularly due to VEGFR-2 suppression. The effects of lenvatinib on renal function have actually received relatively little attention due to the rarity of acute renal injury, but are becoming a new concern for patients on long-term treatment. While several reports have examined the effects of lenvatinib on renal function [6–8], those studies were either case reports or short-term investigations.

The magnitude of urinary protein excretion is recognized as a factor associated with increased risk of progressive renal damage [9] and subsequent end-stage renal disease (ESRD) [10–12]. Iseki et al. reported the ultimate incidences of ESRD among screened individuals with a 17-year follow-up period as 0.2, 1.4, 7.1, and 15.4% among proteinuria-negative, 1+, 2+, and 3+ cases, respectively [9]. This suggests the importance of further investigation of renal outcomes among patients with proteinuria. However, the median observation period of approximately 3 years for lenvatinib in the SELECT trial [13] was far shorter than the period suggested to involve concerns regarding proteinuria in healthy individuals. Our clinical experience [14] showed almost the same prognosis as the SELECT trial. Some patients continue treatment, balancing the degree of disease progression and adverse events (AEs) and the difficulty of proteinuria management [8]. Renal function thus represents a potential new concern when the treatment period is extended in those patients. More than 5 years have passed since lenvatinib was approved for use in patients with advanced differentiated thyroid carcinoma (DTC). The temporal course of renal function and the impact of proteinuria on renal function with long-term lenvatinib exposure have yet to be clarified, with little evidence available on whether lenvatinib induces renal failure. Furthermore, the indications for lenvatinib are now expanding to several cancer types [15–17]. Lenvatinib is metabolized hepatically and excreted renally, so the recommended starting dose differs among types of malignancy. DTC is a cancer type with a low frequency of liver and renal metastases, both of which can affect pharmacokinetics. This study analyzed the long-term effect of lenvatinib on renal function in patients with advanced DTC treated with lenvatinib.

## Patients and methods

### Patients

This study involved DTC patients with the evidence of radioactive iodine-refractory disease who received lenvatinib therapy and who had results available for renal function tests performed at Ito Hospital, Tokyo, Japan, from May 2015 to December 2019. To reveal the long-term renal effects of lenvatinib, patients treated for ≥6 months were investigated. Of the total of 59 DTC patients treated with lenvatinib, 40 (68%) satisfied these criteria and were investigated in this study.

### Management and efficacy of lenvatinib

Lenvatinib was prescribed at a starting dose of 24 mg once daily. Dose interruption or dose reduction in response to adverse events (AEs) was required for treatment continuation. Accordingly, the intensity of treatment was represented as dose intensity (DI), as the average lenvatinib dose in milligrams per day within the treatment period. Morphologic and prognostic treatment efficacy was evaluated.

AEs were assessed based on the National Cancer Institute Common Terminology Criteria for Adverse Events (CTCAE) version 4.0 at every outpatient follow-up, at least every 2 weeks for the first 2 months, then every month thereafter, if the condition of the patient was clinically stable. When treatment-related grade 3 or intolerable grade 2 AEs were encountered, lenvatinib was interrupted until the event in question resolved to grade ≤ 2 or baseline, then sequential dose reductions were implemented if necessary [18].

In addition, proteinuria was assessed based on CTCAE version 4.0, defining: 1+ proteinuria, urinary protein ≥ the upper limit of normal – < 1.0 g/24 h as Grade 1, 2+ and 3+ proteinuria; urinary protein ≥1.0 – < 3.5 g/24 h as Grade 2; and 4+ proteinuria, urinary protein ≥3.5 g/24 h as Grade 3. Instead of a 24-h urine sample, urine protein-to-creatinine ratio (UPCR, g/gCre) was graded based on a previous report confirming its feasibility. After urinalysis performed using a qualitative dipstick test, samples that tested positive (1+ on the dipstick for proteinuria) were sent for UPCR testing the same day [18]. Dose adjustment was decided based on the results of UPCR, as follows: lenvatinib was interrupted for UPCR ≥3.5 g/gCre (i.e., grade 3 proteinuria); and was restarted when proteinuria improved to UPCR < 3.5 g/gCre (i.e., ≤grade 2), as reported previously [18].

Blood pressure (BP) during treatment was controlled mainly using Ca blockers and angiotensin II receptor blockers (ARBs), following the regulatory goal of systolic BP < 120 mmHg, diastolic BP < 80 mmHg in patients without hypertension as a comorbidity and CTCAE grade 1 in patients with hypertension on medication.

### Evaluation of renal function

Estimated glomerular filtration rate (eGFR, mL/min/1.73 m^2^) was calculated as an indicator of renal function. The calculation formulae for eGFR are as follows:

$$ \mathrm{Male}:\left[\mathrm{eGFR}\right]\ \left(\mathrm{mL}/\min /1.73\;{\mathrm{m}}^2\right)=194.00\times \left[\mathrm{creatinine}\right]\ {\left(\mathrm{mg}/\mathrm{dl}\right)}^{-1.094}\times \left[\mathrm{age}\right]\ {\left(\mathrm{years}\right)}^{-0.287} $$

$$ \mathrm{Female}:\left[\mathrm{eGFR}\right]\ \left(\mathrm{mL}/\min /1.73\;{\mathrm{m}}^2\right)=194.00\times \left[\mathrm{creatinine}\right]\ {\left(\mathrm{mg}/\mathrm{dl}\right)}^{-1.094}\times \left[\mathrm{age}\right]\ {\left(\mathrm{years}\right)}^{-0.287}\times 0.739 $$

Values for eGFR were calculated every visit, and data at baseline and 1 month, 3 months, and every 6 months until the 5th year were adopted for evaluation. The adopted data include only until the decision to discontinue treatment was made. Absolute values and change from baseline of eGFR were used for analyses.

Temporal changes in eGFR were investigated for all patients. The definition of renal impairment in this study was set based on these results. Correlations between baseline eGFR and clinical outcomes were investigated.

Furthermore, with reference to Kidney Disease: Improving Global Outcomes (KDIGO) chronic kidney disease (CKD) classifications [19], baseline eGFR was divided into three groups: Group H, high eGFR, defined as ≥90 mL/min/1.73 m^2^; Group M, middle eGFR group, defined as ≥60 but < 90 mL/min/1.73 m^2^; and Group L, low eGFR group, defined as ≥45 but < 60 mL/min/1.73 m^2^. The temporal course of changes in eGFR was analyzed for these three groups. Furthermore, patients were categorized into two groups as Group D (decreased group) and Group ND (not-decreased group) based on the results of time-dependent eGFR changes in all 40 patients. Background characteristics and treatment efficacy were compared between these two groups.

Data up to July 1, 2020 were assessed and retrospectively reviewed.

### Statistics

Statistical analyses were performed using JMP software v12.0 (SAS Institute, Cary, NC). Differences between groups were analyzed using the Wilcoxon test. All *p*-values were two-sided, and values of *p* < 0.05 were considered significant. Survival curves were plotted using the Kaplan–Meier method.

All study participants provided informed consent, and the study protocol was approved by the institutional ethics review committee at Ito Hospital and met the guidelines of our responsible agency. All methods were carried out in accordance with relevant guidelines and regulations.

## Results

### Patients

The background characteristics and renal parameters of patients are shown in Table 1. Median age was 67 years, and 15 patients were male. Five patients showed performance status (PS) 2, all due to bone metastasis. Median baseline eGFR was 72.2 mL/min/1.73 m^2^, and all patients showed eGFR ≥30 mL/min/1.73 m^2^. Six patients had a past renal history of note, including hypertensive nephropathy, drug-induced nephropathy, pyelonephritis, nephrolithiasis resulting in hydronephrotic kidney, and post-nephrectomy status due to malignancy. Renal and liver metastases were detected as of the latest computed tomography (CT) evaluation in 3 and 7 patients, respectively.

**Table 1:** Background characteristics of patients

Patients	40
Age, median [range]	67 [33–78]
Sex, male (%)	15 (37.5)
BW (kg), median [range]	58.0 [35.3–97.1]
Histopathology, follicular (%)	15 (37.5)
Performance status, ≥ 1	17 (42.5)
Sum of tumor diameters (mm), median [range]	82 [11–169]
Tumor related symptoms at baseline, yes (%)	24 (60)
Thyroglobulin doubling time, year	0.7 (0.12–3.0)
Treatment period (months), median [range]	29.5 [6.8–61.5]
Observation period (months), median [range]	32.5 [7.4–61.5]
Dose Intensity, median [range]	9.6 [4.0–16.4]
Best response in RECIST criteria, PR (%)	29 (72.5)
eGFR at baseline (mL/min/1.73 m^2^), median [range]	72.2 (43.6–119.1)
Proteinuria grade 1 at baseline, yes (%)	2 (5)
Past hypertension history (%)	24 (60)
Past diabetes mellitus history (%)	3 (7.5)
Past renal history (%)	6 (15)
Renal metastasis, baseline (%)	3 (7.5)
latest (%)	3 (7.5)
Liver metastasis, baseline (%)	5 (12.5)
latest (%)	7 (17.5)
Proteinuria grade 3, yes (%)	21 (52.5)
Treatment continuation, yes (%)	20 (50)
Treatment termination due to renal problem, yes (%)	7 (17.5)

### Efficacy of lenvatinib

This study excluded patients with short-term treatment (< 6 months), and only patients who could receive continuous, long-term treatment were analyzed. Median DI was 9.6 mg/day. Best response to treatment was partial response (PR) in 29 patients (73%), stable disease in 10 (25%), and progressive disease in 1 (2%), according to RECIST version 1.1 guidelines [20]. Median values for overall survival (OS), time to treatment failure (TTF), and progression-free survival of these patients were 45.4 months (95% confidence interval [CI], 32.4 months–not reached [NR]), 44.1 months (95%CI, 22.5 months–NR), and 19.9 months (95%CI, 14.5–35.3 months), respectively.

Proteinuria was the second most common AE after hypertension. Of the total 39 patients (97.5%) who showed proteinuria, grade 1, 2, and 3 proteinuria was the highest grade in 9, 9, and 21 patients, respectively. Median interval to onset was 12.4 months (95%CI, 0.7–28.5 months). Lenvatinib administration was continued in 19 patients (47.5%) as of the cut-off time. Reasons for lenvatinib discontinuation were deteriorating PS due to disease progression in 14 patients, uncontrollable proteinuria with disease progression in 5 patients, and decreased eGFR in 2 patients (36.6 and 19.9 mL/min/1.73 m^2^, respectively). No patients required initiation of hemodialysis.

### Temporal course of eGFR

**Fig. 1:**
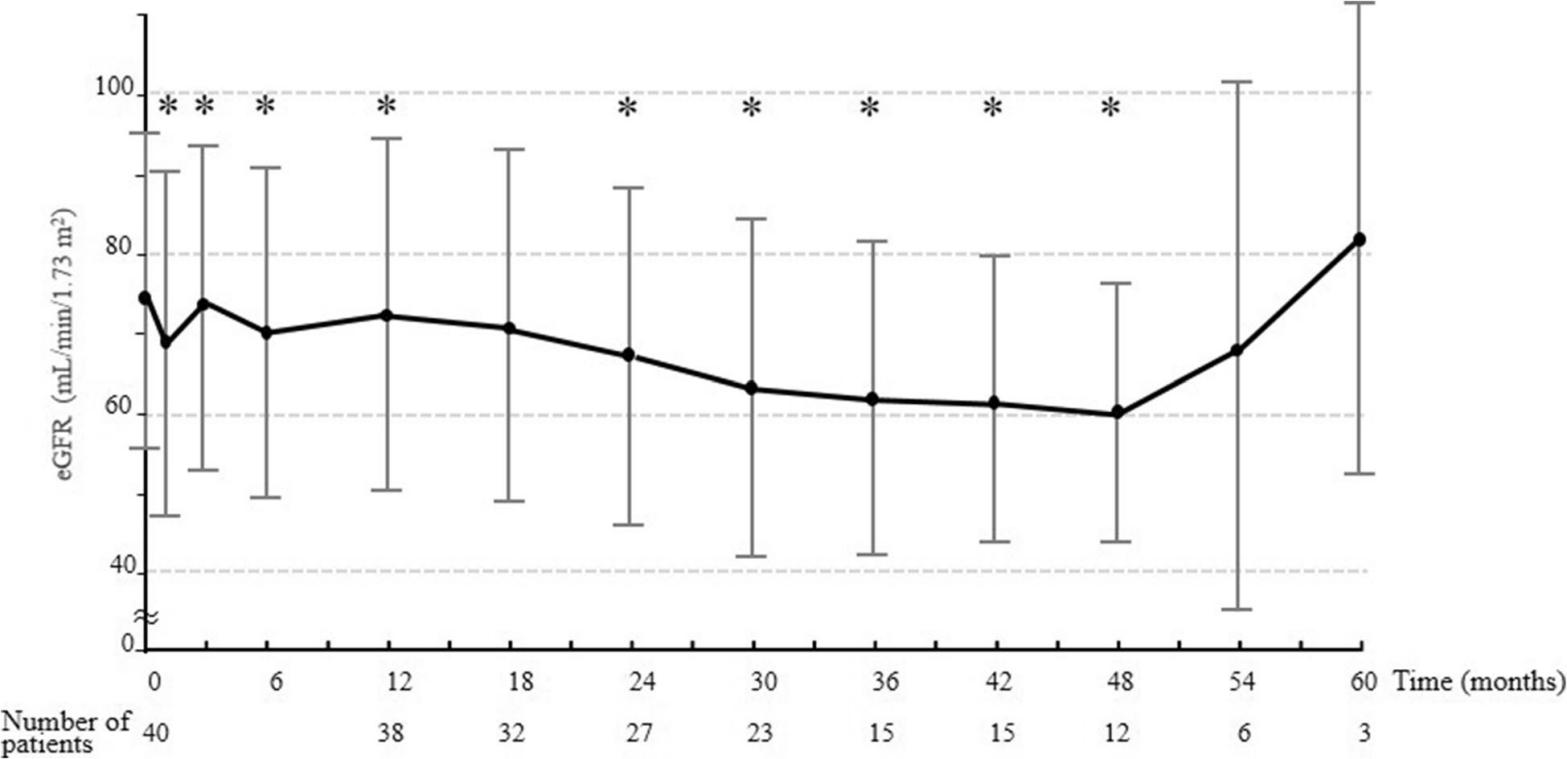
Time course for eGFR changes for the patient population. Mean ± standard *deviation* baseline eGFR for all 40 patients was 73.9 ± 18.1 mL/min/1.73 m^2^. A mild decrease in eGFR is seen over time. Mean eGFR at 12, 24, 36, 48, and 60 months was 70.6 ± 20.9, 66.6 ± 20.5, 61.4 ± 19.3, 59.5 ± 15.7, and 81.3 ± 28.5 mL/min/1.73 m^2^, respectively. *: Time point showing a significant difference compared to baseline eGFR

Courses of changes in absolute eGFR value and changes in value for all 40 patients are shown in Fig. 1. A mild decrease in eGFR was seen over time. Compared to baseline, eGFR at each time point showed significant decreases except for at 18 (*n* = 32), 54 (*n* = 6), and 60 months (n = 3), respectively. Average decreases in eGFR were 11.4, 18.3, and 21.0 mL/min/1.73 m^2^ at 24, 36, and 48 months, respectively. The decreased value reached > 20 mL/min/1.73 m^2^ by 48 months. Median final eGFR was 64.8 ± 22.5 mL/min/1.73 m^2^. Based on the results of time-dependent eGFR changes in all 40 patients, renal impairment in this study was defined a decline in eGFR of > 15 mL/min/1.73 m^2^ continuing ≥6 months, with the final eGFR showing a decrease of > 20 mL/min/1.73 m^2^. Thirteen patients (32.5%) met this definition, and were categorized as Group D.

### Baseline eGFR effect on treatment

**Fig. 2:**
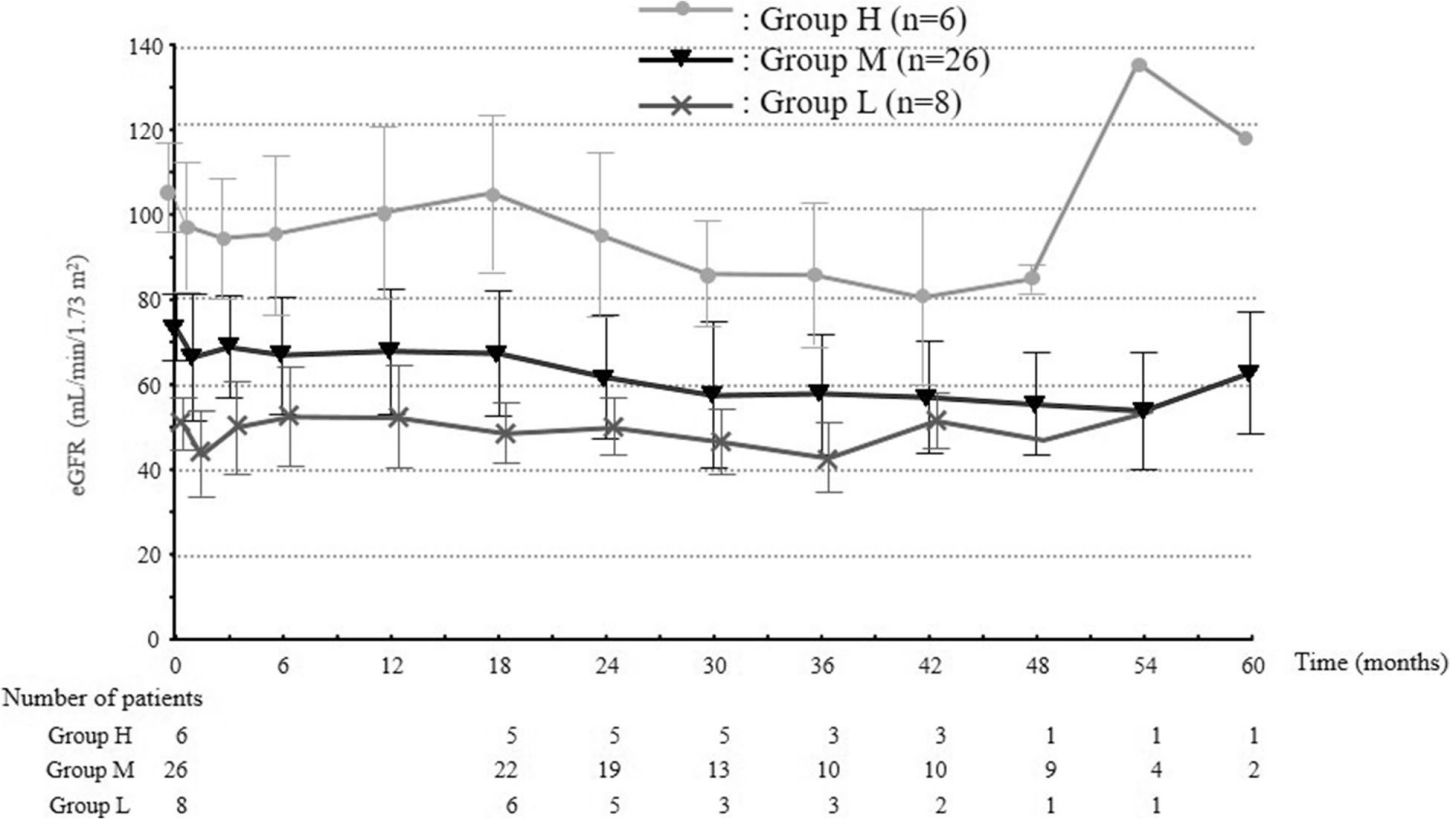
Time course for eGFR changes according to baseline eGFR. Baseline eGFR is divided into the three groups of ≥90 mL/min/1.73 m^2^ (Group H: high eGFR, n = 6), ≥60 but < 90 mL/min/1.73 m^2^ (Group M: middle eGFR, n = 26), and ≥ 45 but < 60 mL/min/1.73 m^2^ (Group L: low eGFR, n = 8). Changes in eGFR over time show no significant differences among these three groups

Background characteristics and treatment efficacy were investigated in the three groups according to baseline eGFR, with 6 (15%) patients in Group H, 26 (65%) in Group M, and 8 (20%) in Group L (Table 2). Older age (*p* = 0.0206), male sex (*p* = 0.0055), and current hypertension (*p* = 0.0207) tended to be associated with low baseline eGFR. Observation period was significantly longer in Group H (*p* = 0.0431). A best response of PR was significantly more frequent in Group H than in other groups (*p* = 0.0463). Temporal changes in eGFR for these three groups were calculated with both the absolute value and the change value (Fig. 2). The eGFR decreased sustainably in all groups, whereas no significant difference in degree of decrease was seen between groups at the same time point.

**Table 2:** Clinical characteristics according to baseline eGFR

Patient	Group H 6 (15%)	Group M 26 (65%)	Group L 8 (20%)	p-value
[background factors]				
Age, median [range]	53.5 [40–70]	66.5 [33–78]	73 [67–78]	**0.0055**
Sex, male (%)	0 (0)	8 (30.8)	5 (62.5)	**0.0206**
BW (kg), median [range]	50.5 [41.6–61]	58.0 [38.5–97.1]	61.5 [35.3–79.2]	0.1810
Histopathology, follicular (%)	3 (50.0)	8 (30.8)	4 (50.0)	0.4916
Performance status, ≥ 1	3 (50.0)	11 (42.3)	3 (37.5)	0.8959
eGFR at baseline (mL/min/1.73 m^2^), median [range]	104.5 [92.8–119.1]	73.3 [60.4–88.7]	49.9 [43.6–58.3]	**< 0.0001**
Proteinuria grade 1 at baseline, yes (%)	0 (0)	2 (7.7)	0 (0)	0.4108
Past hypertension history (%)	1 (16.7)	16 (61.5)	7 (87.5)	**0.0207**
Past diabetes mellitus history (%)	1 (16.7)	1 (3.9)	1 (12.5)	0.4970
Past renal history (%)	0 (0)	3 (11.5)	0 (0)	0.2574
Renal metastasis, (%)	0 (0)	3 (11.5)	0 (0)	0.4108
Liver metastasis, (%)	0 (0)	6 (23.1)	1 (12.5)	0.2255
[treatment factors]				
Treatment period (months), [average]	38.1 ± 16.3	31.3 ± 16.8	29.1 ± 17.7	0.5434
Observation period (months), [average]	46.2 ± 11.8	31.8 ± 15.2	25.7 ± 15.0	**0.0431**
Dose Intensity, median [average]	10.2 ± 2.0	9.4 ± 3.0	10.4 ± 3.5	0.6515
Best response in RECIST criteria, PR (%)	6 (100)	20 (77)	3 (38)	**0.0463**
Proteinuria grade 3, yes (%)	5 (83)	14 (54)	2 (25)	0.0914
Treatment continuation, yes (%)	3 (50)	10 (38)	4 (50)	0.7812
Treatment termination due to renal problem *, yes (%)	1 (17)	5 (19)	1 (13)	0.9020

*: decline of eGFR or uncontrollable proteinuria

**Fig. 3:**
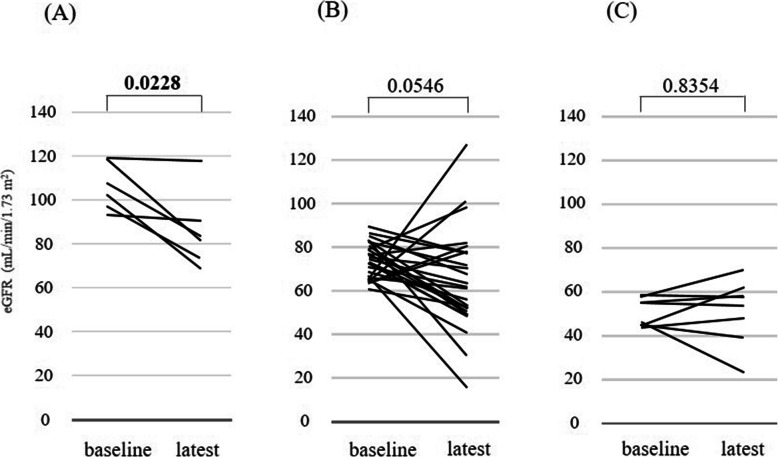
Comparison of baseline eGFR and latest eGFR by baseline eGFR. A) Group H (high eGFR, *n* = 6): eGFR changes from 106.0 mL/min/1.73 m^2^ to 86.1 mL/min/1.73 m^2^, for a mean difference of −20.0 mL/min/1.73 m^2^ (p = 0.0228). B) Group M (middle eGFR, *n* = 26): eGFR changes from 73.8 mL/min/1.73 m^2^ to 64.0 mL/min/1.73 m^2^, for a mean difference of − 9.7 mL/min/1.73 m^2^ (*p* = 0.0546). C) Group L (low eGFR, *n* = 8): eGFR changes from 50.4 mL/min/1.73 m^2^ to 51.3 mL/min/1.73 m^2^, for a mean difference of 0.9 mL/min/1.73 m^2^ (*p* = 0.8354)

Based on the correlation between baseline eGFR and clinical outcome as divided into three groups, values between baseline and latest eGFR were compared (Fig. 3). A significant decrease in eGFR was seen for Group H (*p* = 0.0228), but no significant decreases were evident for Group M (*P* = 0.0546) or Group L (*p* = 0.8345). The latest eGFR values in Groups H, M, and L were 86.1 ± 15.9, 64.0 ± 22.4, and 51.3 ± 13.6 mL/min/1.73 m^2^, respectively.

Lenvatinib was discontinued in Groups H, M, and L due to uncontrollable proteinuria with disease progression in 1, 4, and 0 patients, due to decreased eGFR in 0, 1, and 1 patients, and due to PS deteriorating due to disease progression in 2, 9, and 3 patients, respectively.

### Decrease in eGFR and risk factors

**Fig. 4:**
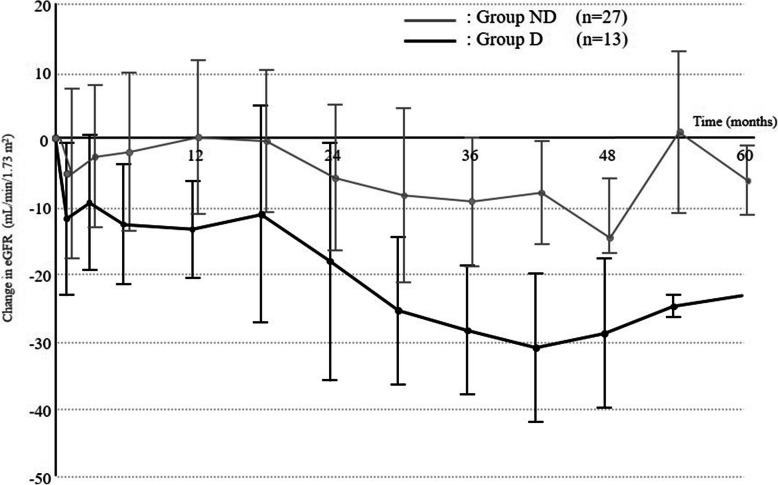
Comparison of eGFR changes between baseline and latest eGFR according to baseline eGFR. Patients are divided into two groups according to degree of eGFR decrease satisfying our definition of renal impairment. Group N (*n* = 13) shows a sustained decrease in eGFR, particularly at 24 months. Group ND (*n* = 27) shows no sustained decrease in eGFR, but tends to show a slight decrease that does not meet our definition

A total of 13 patients (37.5%) who met our renal impairment criteria were labeled as Group D, and the remaining 27 patients (62.5%) as Group ND. Median baseline eGFR was 78.5 mL/min/1.73 m^2^ in Group D and 63.8 mL/min/1.73 m^2^ in Group ND (*p* = 0.0165). A decrease of > 15 mL/min/1.73 m^2^ in eGFR was started at 8.9 months (0.8–37.3 months) in Group D patients. Temporal changes in eGFR in these two groups were calculated with both the change value (Fig. 4) and the absolute value (Table 3). The eGFR of Group D was obviously decreased, since this was defined as the eGFR-decrease group, with decreased values of 18.3, 28.5, and 29.0 mL/min/1.73 m^2^ in months 24, 36, and 48, respectively. Meanwhile, eGFR in Group ND was only slightly decreased, reaching a decrease of > 5 mL/min/1.73 m^2^ after 24 months. The number of patients with baseline eGFR ≥60 mL/min/1.73 m^2^ was significantly higher in Group D than that in Group ND, and was also associated with decreased eGFR (*p* = 0.0072). The long observation period was also associated with a decrease in eGFR, which was considered to indicate that eGFR may decrease in a time-dependent manner. Grade 3 proteinuria was identified as a risk factor for renal impairment (*p* = 0.0283). Of the total of 21 patients with grade 3 proteinuria, 10 patients (47.6%) were allocated to Group D. Of the total of 27 Group ND patients, Grade 3 proteinuria was seen in 3 (11.1%). Clinical factors associated with renal impairment are shown in Table 4. No difference between the two groups was seen in DI calculated as the cumulative dose up to the same time point for each year (Table 5). Lenvatinib was discontinued due to uncontrollable proteinuria with disease progression in 1 and 4 patients, due to decreased eGFR in 1 and 1 patients, and due to PS deterioration resulting from disease progression in 3 and 11 patients in Groups D and ND, respectively. The degree of eGFR decrease in 1 patient discontinued due to eGFR decrease in Group ND was not compatible with our definition.

**Table 3:** Changes in eGFR value according to renal function decrease

Observation period, months	Group D				Group ND			
	Number of patients	Absolute value, (mL/min/1.73 m^2^)	Changed value, (mL/min/1.73 m^2^)	p-value	Number of patients	Absolute value, (mL/min/1.73 m^2^)	Changed value, (mL/min/1.73 m^2^)	*p*-value
0	13	83.9 ± 16.2	–	–	27	69.6 ± 17.0	–	–
1	13	71.9 ± 20.9	−12.0 ± 11.4	0.0033	27	63.5 ± 2.9	−5.3 ± 12.6	0.0403
3	13	74.2 ± 19.9	−9.6 ± 9.9	0.0057	27	67.1 ± 16.1	−2.8 ± 10.6	0.1874
6	13	71.0 ± 22.3	−12.9 ± 8.9	0.0003	27	67.9 ± 17.2	−2.1 ± 11.7	0.3697
12	13	70.3 ± 18.7	−13.5 ± 7.1	< 0.0001	25	71.2 ± 21.9	0.1 ± 11.4	0.9605
18	13	72.5 ± 25.4	−11.4 ± 15.9	0.0295	19	68.8 ± 19.1	−0.47 ± 10.6	0.8533
24	12	66.6 ± 25.5	−18.3 ± 17.6	0.0009	15	66.6 ± 15.3	−5.93 ± 10.7	0.0571
30	11	59.8 ± 22.0	−25.6 ± 11.0	< 0.0001	10	65.8 ± 18.1	−8.5 ± 12.9	0.0791
36	7	54.9 ± 16.6	−28.5 ± 9.4	0.0003	8	67.1 ± 19.6	−9.4 ± 9.5	0.0346
42	7	52.3 ± 13.5	−31.1 ± 10.9	0.0004	8	68.4 ± 17.3	−8.1 ± 7.6	0.0252
48	6	52.3 ± 15.3	−29.0 ± 11.0	0.0020	6	66.7 ± 12.6	−11.5 ± 5.4	0.0136
54	3	46.3 ± 6.4	−25.0 ± 1.7	0.0022	3	88.4 ± 34.6	0.9 ± 11.9	0.9276
60	1	48.6	−23.4	n.c.	2	97.6 ± 20.4	−6.3 ± 5.2	0.4393

*n.c.* not calculated

**Table 4:** Clinical factors for renal function decrease

Patient	Group D 13 (37.5%)	Group ND 27 (62.5%)	*p*-value
[background factors]			
Age, median [range]	66 [46–76]	69 [33–78]	0.1650
Sex, male (%)	2 (15.4)	11 (40.7)	0.0951
BW (kg), median [range]	57.9 [41.6–74.0]	60.7 [35.3–97.1]	0.4356
Histopathology, follicular (%)	5 (38.4)	10 (37.0)	0.9306
Performance status, ≥ 1	6 (46.5%)	11 (40.7%)	0.7460
eGFR at baseline (mL/min/1.73 m^2^), median [range]	78.5 [65.2–118.7]	63.8 [43.6–119.1]	**0.0165**
eGFR at baseline (mL/min/1.73 m^2^), < 60	0 (0)	8 (29.6)	**0.0072**
Proteinuria grade 1 at baseline, yes (%)	2 (15.4)	0 (0)	**0.0298**
Past hypertension history (%)	8 (61.5)	16 (59.3)	0.8903
Past diabetes mellitus history (%)	1 (7.7)	2 (7.4)	0.9745
Past renal history (%)	1 (7.7)	5 (18.5)	0.3452
Renal metastasis, (%)	0 (0)	3 (11.1)	0.1158
Liver metastasis, (%)	1 (7.7)	6 (22.2)	0.2296
[Treatment-related factors]			
Treatment period (months)	34.1 [12.8–60.5]	25.2 [6.8–61.5]	0.0832
Observation period (months)	45.4 [21.0–60.5]	27.2 [7.4–61.5]	**0.0115**
Dose intensity (mg/day), median [range]	9.7 [4.1–14.0]	9.46 [4.0–16.4]	0.7398
Best response in RECIST criteria, PR (%)	10 (76.9)	19 (70.4)	0.6606
Proteinuria grade 3, yes (%)	10 (76.9)	11 (40.7)	**0.0283**
Treatment continuation, yes (%)	8 (61.5)	11 (40.7)	0.2161
Treatment termination due to renal problem, yes (%)	2 (15.4)	5 (18.5)	0.8053

**Table 5:** Dose intensity according to decrease in renal function

	Group D (*n* = 13)		Group ND (*n* = 27)		*p*-value
Initial dose (mg)	24				
Time (months)	n	DI (mg/day)	n	DI (mg/day)	
12	13	11.9 (7.6–13.7)	27	11.6 (4–18.1)	0.7507
24	13	9.7 (6–14)	24	9.8 (6.7–16.4)	0.4640
36	10	9.2 (5.2–13)	14	9 (7.2–13.2)	0.8836
48	6	8 (4.5–10.5)	7	8.7 (6.3–10.1)	0.7751
60	4	7.4 (4.1–10.5)	6	9.05 (5.6–9.8)	0.6698

## Discussion

This investigation was conducted to clarify the long-term effects of lenvatinib on renal function. VEGF is an essential factor for glomerular structure [21], and this study was supported by the fact that VEGFR-suppressing agents such as lenvatinib can induce proteinuria [1–4, 6]. Lenvatinib is indicated at present as a monotherapy in patients with radioiodine-refractory DTC [13] and unresectable hepatocellular carcinoma [15]. Further indications are expected [16, 17]. The recommended initiation dose of lenvatinib differs according to the type of malignancy. DTC is a cancer type with a low frequency of liver or renal metastases, which can affect drug metabolism and excretion in rare cases. The results of this investigation could provide insights into the treatment of other malignancies.

Overall, renal function decreased over time to a relatively small degree within 2 years, then declined continuously thereafter. Renal impairment in this study was uniquely defined as a decline in eGFR of > 15 mL/min/1.73 m^2^ for ≥6 months, with a total decrease of > 20 mL/min/1.73 m^2^ as of the latest eGFR. Approximately one-third of patients met the definition of renal impairment, confirming that lenvatinib can affect renal function. The international definition of chronic kidney disease (CKD) is a glomerular filtration rate (GFR) < 60 mL/min/1.73 m^2^, or markers of kidney damage, or both, for ≥3 months, regardless of the underlying cause. Unlike that general definition, a slight eGFR decrease during cancer therapy regardless of baseline eGFR can be detected by our definition. Adopting this definition as a valid indicator for recognizing that eGFR is starting to decline can trigger closer attention to renal function. With this definition, the comparatively acute renal impairment due to end-stage cancer that results in deterioration of whole organs can be differentiated from renal impairment induced by lenvatinib. Conversely, short-term declines in eGFR due to lenvatinib cannot be detected using this definition, but such declines are rare. Distinguishing between these two factors is also difficult in patients with end-stage cancer.

The observation period was significantly longer in Group H among the three groups divided by baseline eGFR. The change between baseline and latest eGFR was significantly different in Group H (Fig. 3). Furthermore, no differences in degree of decrease were seen among the three groups at the same time point (Fig. 2, Table 2). That is, the degree of decline in eGFR was unaffected by baseline eGFR. This suggests that no special attention needs to be given to renal function when baseline renal function is acceptably low (e.g., eGFR ≥45 but < 60 mL/min/1.73 m^2^). This also suggests that patients with high renal function have abundant reserve, resulting in an ability to continue treatment for longer. This is supported by the fact that the rate of RECIST-PR and frequency of proteinuria were highest among patients in Group H, and that neither PS at baseline nor renal reason for lenvatinib discontinuation differed significantly between groups. From these assessments, although not definitive, eGFR at baseline is not considered a prognostic predictor as much as a predictor of tolerance for AEs.

When patients were divided into two groups according to the presence or absence of renal impairment, a marked decrease in eGFR was certainly seen after 2 years in Group D. A mild decline was seen even in Group ND, although the degree did not meet the definition (Fig. 4, Table 4). Lenvatinib can thus induce renal impairment in some patients, increasing the potential for deterioration over time. No involvement of DI in the same observation period was seen (Table 5).

Proteinuria was revealed to increase the risk of renal impairment. This may indicate the same phenomenon, i.e., the increased risk of ESRD in a span of > 10 years, seen healthy subjects with proteinuria, but over a shorter time span. Still, of the 21 patients with grade 3 proteinuria, only 10 patients (47.6%) showed a decrease in eGFR, whereas even among the 19 patients without grade 3 proteinuria, 3 patients (16.0%) showed a decrease in eGFR. Proteinuria may be just one phenotype of renal damage caused by VEGFR inhibitors, and even patients without proteinuria should be aware of the potential for changes in renal function.

Patients with baseline grade 1 proteinuria appear able to receive treatment safely for a long time regardless of the appearance of proteinuria. Since this pathology is not high-grade proteinuria equivalent to grade 3, these patients were examined together with cases showing no proteinuria in this study.

Proteinuria is managed continuously with lenvatinib DI regulation while looking at the balance with disease control. Meanwhile, renal impairment cannot be immediately improved just with regulation of the lenvatinib dose. Once ultimate renal impairment occurs, treatment must be suspended irrespective of successful disease control. Although no patients required initiation of dialysis in this study, eGFR could logically decrease enough to require dialysis over a long treatment period. The timing of a change to the next treatment line is thus the next important clinical question [8], but that issue cannot be addressed using the present results. Only limited lines of treatment are available for DTC, unlike for some other malignancies. Where multiple treatment options are available, treatment with one agent does not need to be prolonged when eGFR is decreasing. Sorafenib, another agent approved for DTC, rarely induces proteinuria [8] and was confirmed as safe by Tatsugami et al., albeit in a 1-year investigation [22, 23]. Dialysis can directly affect quality of life. Ideally, the decision should be made in advance regarding whether dialysis should be initiated when renal function finally fails, in accordance with recommendations from the field of onconephrology [24, 25]. Prolongation of OS with anti-cancer treatment is obviously given very high priority [14], along with consideration of renal prognosis commensurate with the oncological prognosis in patients receiving lenvatinib. The balance between acceptable risk of harm and potential benefit from lenvatinib treatment is an important aspect of therapy [14].

In our study, recovery of renal function after lenvatinib discontinuation was not able to be discussed due to insufficient data from patients after lenvatinib cessation.

Two key limitations to this study should be considered. First, this analysis was limited to Japanese patients. This population reportedly shows a high frequency of proteinuria induced by lenvatinib compared to all subsets, including other ethnicities [13, 15, 26]. The smaller number of nephrons may be related to this phenomenon, although the details have yet to be clarified [27]. Ethnicity-specific renal effects of lenvatinib also remain unclear. The DI for Japanese populations in the real world may tend to be lower than DIs reported from other countries, and the relationship of a high frequency of proteinuria with this point is also unclear. The second limitation was the lack of consideration given to the muscle mass of each patient. As eGFR is an index using serum creatinine level, values are affected by muscle mass. Since some patients receiving treatment may have had sarcopenia [28], eGFR may have been overestimated in cachexic patients.

To the best of our knowledge, this is the first study to describe the long-term efficacy of lenvatinib on renal function in patients with advanced DTC treated with lenvatinib in actual clinical practice.

Our study revealed that lenvatinib can induce renal impairment, especially in treatment periods > 2 years, regardless of baseline eGFR. Lenvatinib can be used safely, at least in terms of renal effects, for periods within 2 years. Patients who start therapy with better renal function have a larger standby capacity, allowing longer clinical application. Grade 3 proteinuria is a risk factor for renal impairment. Decreased eGFR does not necessarily warrant immediate treatment discontinuation, and ideally treatment continuation should be decided according to the balance between acceptable risk of harm and potential benefit from lenvatinib.

## Data Availability

All data generated or analyzed during this study are included in this published article.
